# On Chronic Poisoning with Chloral Hydrate

**Published:** 1873-08

**Authors:** Ludwig Kirn


*On Chronic Poisoning with Chloral Hydrate.* By DR. LUDWIG KIRN. (From the Allgem. Zeitsch. fur Psychiatrie.) Translated and condensed by the Editor of *The Practitioner*.

The immense literature of chloral hydrate, which represents, however, but a fraction of the cases actually observed, affords many references to acute poisoning, involving the greatest danger to life, or ending fatally in spite of remedies. Cases of the latter kind have appeared, especially in the English journals, with a truly fearful frequency.

Much fewer are the published observations on the changes which the long-continued use of chloral hydrate produces in the organism. If some observers have failed to recognize any such changes even after long administration, it should be remembered that not only are the slighter disturbances liable to be overlooked, but especially that, from the experience of ourselves and others, the individual tolerance of patients for chloral varies within very wide limits.

We administered chloral hydrate, for the most part, once daily, as a hypnotic, in the dose of 30 to 60 grains, or occasionally 75 to 90 grains; with our present experience we should now rarely go beyond the dose of 30 grains. In particular cases we gave, besides the evening dose, one or two others in the day, to calm excitement. Our early experiments having warned us against the hypodermic use of chloral, it was afterwards always given by the mouth (or else by the rectum, with similar effects).

The calming and hypnotic action of the drug in cerebral and nervous disorders proved to be exceedingly variable; whilst with many the commencing dose of 30 grains continued for a long time to produce the desired effect, and in others only a quite gradual increase of this quantity was needed, in other patients there was but slight result, although the dose was quickly increased to 60; and in one case no sleep was obtained, although the amount was rapidly raised to 90 grains. In many patients, under apparently similar external conditions, the same quantity would sometimes succeed and sometimes fail. We shall here only concern ourselves with a series of morbid phenomena which appeared sooner or later in the course of a continuous employment of this hypnotic, collating these with the experiences of others.

The most prominent of the symptoms were those connected with the skin; their production for the most part depended on the cutaneous vaso-motor nerves. They were more or less extensive erythemas, and pustular or papular exanthemata.

The clinical observations of Schule* on chloral erythema must here take the first place. "The use of chloral produces a tendency to fluxionary hyperæmias, with increased and strengthened heart-action. This is first and most considerably manifested in the head, the vessels being dilated, and an intense erythema occurring, at first in spots, but afterwards more diffusely. In the more pronounced forms the erythema extends downwards to the trunk, and becomes general, in which case it seems to follow, by preference, the course of the larger nerve-trunks. This chloral-rash remains latent until it is set going by some stimulus to the vascular system, but then appears in an intensity and rapidity which are proportioned to the existing current of (general) chloralization."

* Ueber eine bemerkenswerthe Wirkung des Chloral-Hydrats. Allgem. Zeitsch. fur Psych. xxviii. 8.

I reproduce these results of Schule's verbally, because I can entirely confirm them. Although, from the varying chloral idiosyncrasies of individuals, they are not always observed, the statement is true for a great number of cases, and I have at this moment a series of patients under my eyes in whom the chloral-rash can be produced with the certainty of an experiment. For instance, in a paralyzed patient, who took 30 grains of chloral every night, ten minutes after she had drunk her beer, there occurred increased action of the heart and spots of roseola upon the forehead, nose, cheeks, and neck, which quickly coalesced into a patchy erythema, with swelling and heat of the affected parts, which symptoms disappeared in about an hour. Still more strongly appeared the same symptoms in a young and previously healthy woman, affected with mania, who every night took 30 to 45 grains of chloral (although she had not previously suffered from congestion of the face). As soon as she took a glass of beer there was strong pulsation of the arteries, and the whole face was swollen and of such an alarmingly deep color that we were obliged to forbid the use of wine and beer. At present we but rarely allow alcoholic drinks to patients who are being treated with chloral. In another young woman suffering from mania, who took 30 grains of chloral at night, four ounces of wine were sufficient to induce the chloral-rash.

Chloral erythema has also been studied from other sides; we may specially mention the observations of C. Browne, which led him independently to results similar to those of Schule. He too observed, as a consequence of chloral, a great tendency to congestions of the head and face, which in a few cases were limited to the cheeks, but in many extended to the forehead, neck and ears. He observed simultaneous excitement of the vascular system, strong contraction of the pupils, and ingestion of the conjunctiva. The symptoms also were at their height after the taking of quite moderate doses of alcohol; he attributed this to temporary paralysis of the vaso-motor centres in the head and neck. Browne also observed another case in which, after a dose of chloral (the amount of which is not stated), a diffuse inflammatory redness appeared over the whole body, so that it was thought advisable to isolate the patient; in ten hours this redness had disappeared. Husband gave to a patient daily for eight days, two 20-grain doses, and for five days more two 30-grain doses of chloral; a scarlatinal rash broke out over the whole body, accompanied by fever and tenderness of the skin, and was followed by desquamation.

The effect is not always limited to congestion and erythema of the skin, but other skin affections are occasionally produced; for instance, our female patients, who had taken 60 grains of chloral daily, first had erythema of the face, and later a papular rash on the arms, with red bases. In some, nettle-rash occurred.

The swelling, accompanied or not by rash, which has been noticed in the face after continued use of chloral, may equally occur on extensive portions of the body. In several patients observed at Illenau, a swollen condition of almost the whole body was noticed, which might be ascribed to serous infiltration of the skin from stasis of blood.

With these affections of the skin must be associated affections of the mucous membranes, which, so far as they are not the result of direct chemical irritation, must be considered analogous to the former; *i. e.*, they are produced by paralysis of the vaso-motor centre. For instance, in some patients treated with chloral we observed redness, swelling, and secretion of the conjunctiva, which became severe if the medicine was continued, but speedily disappeared on its disuse. Other observers, especially Balfour and Brady, have noticed this conjunctivitis. In three of our patients, probably from the mechanical irritation of the medicine, there was moderate catarrhal sore-throat, with pain in the fauces. In one of Curschman's patients there was pronounced redness and swelling of the epiglottis and of the false vocal cords.

The evil effects of chloral are not always limited to vaso-motor paralyses and transitory neuroses of the skin and mucous membranes; if the conjestions are not removed, the after-consequences may be severe or even dangerous if the seat of the affection is in the vital organs. We may here refer to a case which resembles the former as regards the affection of the skin and mucous membrane, but which became serious in consequence of severe swelling of the glands (Chapman: *Lancet*, 1871). Reimer observed in a series of patients the following fact, especially after the use of morphia and chloral together: After slight external pressure there was congestion in circumscribed spots with much lowered sensibility, which quickly disappeared if the pressure was soon removed. Under less favorable circumstances, the red spots swelled and assumed a darker color; vesicles were soon developed, which might even run on to sloughing. This phenomenon is distinguished from the usual mark after long pressure in conditions of gradually sinking vitality, especially because, whilst the epidermis and cutis remain intact, it extends into the deep subcutaneous tissue.

An important symptom which we have noted in a series of cases of the long-continued use of chloral is an interference with respiration, which may remain slight, and scarcely troublesome to the patient, or may become positive dyspnœa. This symptom was experimentally produced by the Swede Hammersten, who observed severe dyspnœa in cats that had taken chloral, and was briefly noticed by Jastrowitz, one of whose patients, while taking chloral, suffered from severe dyspnœa with occasional cessation of breathing; and it was finally completely described and explained by Schule, who observed a patient who, after long use of chloral, used regularly to suffer after meals from a sense of oppression which made going up stairs extremely difficult, and even interfered with speech, although there was no chest disease to account for this. The symptoms persistently recurred in spite of all treatment till the chloral was left off, when the oppression entirely disappeared.

A similar chloral dyspnœa, though not so long continued, occurred in many cases observed by us, either with or without a rash, and a feeling of heaviness and anxiety.

That the chloral dyspnœa does not always stop at the lower degrees, but may proceed to the most severe and dangerous developments, is shown by the following observation communicated to me by an eminent physician. This gentleman was summoned in consultation to a lady prostrated by long sufferings, who had of late suffered from attacks of extreme dyspnœa, which had increased to asphyxia; at the same time the face was swollen, the facial muscles paralyzed, and there were also all the signs of cerebral effusion. Every remedy had failed, and the patient seemed on the brink of the grave. The physician therefore recommended the discontinuance of a daily dose of 45 grains of chloral which had been given as a hypnotic; whereupon all these highly alarming symptoms vanished in an almost magical way, the cerebral disturbance ceased, and the respiration quickly resumed its normal type!

The dyspnœa may be anatomically explained, by analogy with the effects of chloral upon the skin and mucous membrane, by hyperæmia of the lungs which is produced through the channel of the vaso-motor nerves. We find here a further confirmation of the assumption that chloral operates upon the vaso-motor centre and the medulla oblongata, and that its paralyzing influence extends thence to the peripheral branches of the affected nerves. This might also lead to a practical contra-indication of chloral in all morbid conditions where there is a tendency to congestions or stases of blood in the lungs.

We come now to a fourth group of cases in which both the quality of the symptoms and their greater or less extension in the organism indicate a distinct change in the composition of the blood. In this connection the following cases observed by Crichton Browne may first be referred to; their interesting phenomena justify a detailed report:—

Case I.—A woman, aged 69, suffering from periodical mania, had 20 grains of chloral thrice daily; on the fourth day a redness was developed on the skin of the chest and shoulders, which did not vanish on pressure; on the sixth day the eruption had extended over the whole trunk and limbs, livid spots and deep red patches alternating. The lips and the mucous membrane of the mouth were excoriated, the gums spongy, the tongue blistered and ulcerated, the breath fœtid. The general state was one of great depression; pulse 120. On the eleventh day the ulceration of the mouth had extended further; the lips were covered with crusts. The petechial eruption was diminished on the chest and abdomen, the spots were yellowish, with patches of white skin between them; the spots on the arm lost their redness later. On the fifteenth day there was a sort of general desquamation; fissures of the skin over the sacrum, and in the neighborhood of the joints. From that time convalescence proceeded, and ordinary health was restored.

Case II.—A woman, aged 46, suffering from cardiac disease, hemiplegia, and dementia, took 15 grains of chloral three times a day, with a calming effect. On the nineteenth day of the treatment numerous purple-red spots appeared in the neighborhood of the left elbow; on the next day many similar spots were seen on the shoulders and fore-arms, which coalesced with the others. On the twenty-first day livid spots came on the face; the left arm swelled and became hard; on its reddened surface appeared a multitude of minute points of a much deeper color, which did not diminish on pressure. Next day there were dark purple spots and discolorations, some small, round, and circumscribed, others broad and irregularly shaped, on the legs and abdomen, and in stripes on either side of the vertebral column. Simultaneously with the petechiæ there were great prostration, tendency to somnolence, weakness and excitability of the pulse, sore lips, thickly coated tongue. On the twenty-third day the spots and discolored patches had extended in every direction, and the previously bright red spots had assumed a deep purple color. Finally, signs of lung congestion appeared, with gradual failure of power, and death, after several fainting-fits, on the twenty-sixth day. At the autopsy numerous ecchymoses of every shape and size were observed more or less on all parts of the skin; the right lung was congested and œdematous, the heart dilated and its valves thickened; over the right central hemisphere there was a large arachnoid cyst containing fluid blood.

With the foregoing may be joined a case related by Monkton, in which, after four days' administration of 60 grains of chloral daily, a rash resembling slight variola with hæmorrhagic purpura appeared, and death occurred on the sixth day, by syncope. Finally may be mentioned two patients of Pelman, in whom, after treatment with chloral, there were larger and smaller petechiæ over the whole skin: in one, which proved fatal, numerous petechiæ were found on the mucous membrane of the larynx and under the endocardium; and a hæmatoma on the right side in the skull reaching to the base, the fluid contents of which gave evidence of their recent origin.

I shall now relate a case observed by myself, which is yet more striking from the multiplicity of its phenomena, which are of a kind perhaps to give us some clearer understanding of their origin. The case was observed by me at a time when the evil effects of chloral hydrate were not yet known; the medicine was therefore continued in spite of the most multiplied symptoms, because chloral was not for a long time recognized as their cause: the affection was therefore followed up further than we should dare to do now that our increased knowledge would oblige us to stop at an earlier stage. [Here follows a detailed report of the case, which need not be given.] If we sum up the weightier symptoms of this case, we find a young, strong, previously healthy person suffering from uncomplicated mania, in whom, on the ninth day of chloral treatment, a rash appeared in the form of groups of red spots, which soon became confluent. On the twentieth day the temperature and pulse rapidly rose to a febrile pitch; three days later the temperature had reached 106.7°; large and repeated doses of quinine were given without result, and baths had only a temporary effect. Œdematous swelling of the face, cheeks, eyelids, and ears now set in. During the whole course of the disease, the skin, so far from returning to its natural appearance, was the seat now of impetiginous, now of moist, and now of scaly eczema and ichthyoses, so that the process of desquamation, instead of being short as in the acute exanthemata, occupied many weeks, during which great sheaths of epidermis were cast off from all parts of the body. The profound lesions of the skin nutrition were evidenced in the later stages by a remarkable shedding of the hair and a gradual falling off of all the nails of the hands and feet. The affection of the skin was accompanied by a similar one of the mucous membranes, first of the intestines, which kept up watery diarrhœa in spite of medicines, and then by a similar affection of the conjunctiva and the bronchi. From the sixth week of the disease onwards a series of large abscesses formed on both arms over the shoulders and arm-pits, which secreted a considerable quantity of pus. Whilst these phenomena were occurring, there had been for eight weeks a continuous fever, occasionally remitting, and then again running up to a temperature beyond 104°.

The symptoms which we have now collectively described must be defined as chronic blood-poisoning. We cannot, however, place this in any of the known groups; we have not to do with a pyæmia or septicæmia, nor with a metallic or vegetable poisoning, since none of the causes have been at work which would lead to these affections, nor do we observe their characteristic phenomena; still less did the affection which the medicine produced in this patient resemble puerperal mania. In fact, there was no other external cause except the administration of the chloral: this medicine, which in even much larger single doses produces no such effect, was for ten weeks given in nightly doses of 45 to 60 or even 75 grains, sometimes in two doses daily. The symptoms began, after a certain saturation had been produced by accumulation, to spread further and further, and finally to assume the complete picture of a chronic blood-poisoning.

The origin of the disease leads us thus, by exclusion, to conclusions which have a high degree of probability, and we are also in a position to adduce positive facts. If a glance be cast at the symptoms observed by ourselves and others, after a more or less continued administration of chloral, we meet with the greater part of the phenomena observed in our last case, especially the very various affections of the skin and mucous membranes, the alterations in vascular action, and finally the profound alterations of the blood, which in some cases remind us of the phenomena of scurvy. The characteristic feature in the morbid picture which we have given consists less in symptoms which in themselves are altogether new, than in the assemblage of the most heterogeneous phenomena, which previously had only been observed singly, in one person and in a most aggravated degree of intensity.—*The Practitioner.*

